# A comparative study on the Antihyperlipidemic and antibacterial potency of the shoot and flower extracts of *Melastoma malabathricum* Linn's

**DOI:** 10.1186/s40816-023-00355-6

**Published:** 2023-03-01

**Authors:** Md. Abdul Kader, Md. Masuder Rahman, Shahin Mahmud, Md. Sharif Khan, Shamsunnahar Mukta, Fatama Tous Zohora

**Affiliations:** 1 Department of Bitechnology and Genetic Engineering, Mawlana Bhashani Science and TechnologyUniversity, Santosh, Tangail-1902, Bangladesh; 2 Department of Plant and Environmental Biotechnology, Faculty of Biotechnology and Genetic Engineering, Sylhet Agricultural University, Sylhet-3100, Bangladesh

**Keywords:** *Melastoma malabathricum*, Antibacterial, Hyperlipidemia induction, Anti-hyperlipidemic, Histopathology

## Abstract

**Background:**

Atherosclerosis is arteries’ thickening and stiffening condition manifested due to plaque formation by oxidized-LDL of abundant and deranged lipid metabolism. Traditionally, *Melastoma malabathricum* Linn (MM) leaves are used for anti-diabetics, abdominal problems, and high blood pressure. The current experiment unveils the potency of ethanol, acetone, and water MM extracts as antibacterial agents and alternative medicine during hyperlipidemic conditions.

**Methods:**

A high cholesterol diet (HCD-2500 mg/kg) was provided with regular feeds for 3 weeks to induce hyperlipidemic mice. Afterward, comparing weight with Group-A (normal control), the hyperlipidemic mice were classified into five groups: Group-B (hyperlipidemic control), Group-C (MFA-500 mg/kg), Group-D (MSE-250 mg/kg), Group-E (MSE-500 mg/kg), and Group-F (ATOVAT-20 mg/kg). And the dosages were given orally for 28 days according to their body weight. Fasting blood was collected at the end of treatment, and serum was taken to test lipid profiling and liver enzymes.

**Results:**

The body mass had waxed significantly (*P* < 0.001) in all the groups compared with Group-A. Subsequently, orally administered different doses where group-D and group-E demonstrated magnificent anti-hyperlipidemic potency (*P* < 0.001) compared with group-B. During treatment, rapid upward body mass was tardy in group-E (*P* < 0.001). However, the liver enzyme expression such as AST, ALT, and ALP was elevated (*P* < 0.001) in Group-F, they were significantly lessened (*P* < 0.001, *P* < 0.01) in Groups-C, D, and E, which indicates these extracts have significant anti-liver damaging potency. Alongside the antibacterial activity of MSE-1500 μg/disc, it exhibited the greatest (16.50 mm) zone of inhibition against *Shigella dysenteriae.*

**Conclusion:**

However, in our current experiment, depending on the derived data, we can elicit that the *Melastoma malabathricum* shoot ethanolic (MSE) extract is a potential resource for developing alternative medicine to manage the hyperlipidemic condition.

## Introduction

Hyperlipidemia is a chronic propulsive complication resulting from the aberrant metabolism of carbohydrates, proteins, and fats. It nestles as elevated levels of fat molecules such as Total cholesterol (TC), Triglyceride (TG), Low-density lipoprotein (LDL), and Very low-density lipoprotein (VLDL) in the circulatory system [1]. The fundamental cause of hyperlipidemia is lopsided energy existing in the bloodstream between calories consumed and calories expenditure which is bolstered by a sedentary lifestyle [2]. Alongside, the augmented blood cholesterol can be manifested due to genetic reasons [3]. The progressed hyperlipidemic condition is intertwined with several severe maladies in the body such as cardiovascular disease (CD) [4], diabetes mellitus type 2 (T2DM) [5], non-alcoholic fatty liver disease [6], kidney disease, reproductive disease as well as osteoarthritis [7]. In 2019 approximately 17.9 million people died from CD, representing 32% of all global deaths in which 85% had succumbed because of heart attack and stroke. In the impending days, experts are conjecturing that the prevalence of CD will be prodigious due to sedentary lifestyle during the covid-19 pandemic and infectious nature of coronavirus [8].

Cardiovascular diseases such as heart failure (FR), peripheral arterial disease (PAD), and coronary heart disease (CHD) evolve because of deposit fatty tissue (Plaques) inside the blood vessel called atherosclerosis [9]. As a result, the blood flow is averted to reach several organs such as the heart, brain, and extremities that belong to heart attack, stroke, and peripheral arterial disease. Atherosclerosis is inflammation triggered by the oxidized-LDL molecules propelled by inflammatory cytokines and biomarkers [10]. It has been recorded recently the production of reactive oxygen species (ROS) engendered as a byproduct of aerobic metabolism, drug, and toxins are entangled with many human diseases such as cardiovascular disease, cancer, Alzheimer’s disease (AD), aging, and atherosclerosis [11, 12].

The magnificent contribution of phytochemicals is combating several diseases such as cardiovascular [13], Cancer, Colorectal, diabetics, and Bacterial disease [14]. They also exhibit antioxidative (especially flavonoids) properties to mitigate the ROS to escape these detrimental diseases [15]. Where xenobiotics have a drastic impact on the liver and kidney during the treatment of diseases, the phytochemicals have impressive results without any side effects. This penchant propelled us to quest for anti-hyperlipidemic medicinal plants that produce several phytochemicals in leaves, flowers, roots, and fruits. These phyto-compounds act on various mechanisms to cure cancer, cardiovascular and bacterial disease [16]. During biotic and abiotic stresses, plants produce a plethora of secondary metabolites such as polyphenols, flavonoids, terpenoids, alkaloids, and plant sterols to defend themselves and provide unique bioactivity on humans health [17].

The Small shrub *Melastoma malabathricum* Linn (MM) belongs to the family of Melastomataceae commonly available in tropical and temperate Southeast Asian countries, locally known as Phutki to Bangladesh, India, and Senduduk to Malay [18]. Its leaves, shoots, barks, and roots are processed in various ways to treat various types of diseases such as high blood pressure, diabetes, dysentery, diarrhea, piles, leucorrhea, cancer, epilepsy, ulcers, gastric ulcers, skin diseases, arthritis, tenderness in the legs, bleeding, toothache, and smallpox from the traditional times [19].

The *Melastoma malabathricum* Linn (MM) leaves have traditionally been used against different diseases, but no comparative study of ethanol and acetone extract is available in the hyperlipidemic condition. Alongside, the relative study of ethanol and water extract against enlisted pathogenic bacteria in our current investigation was undocumented. That’s why to stand alternative medicine; an attempt was taken to unveil the unique potency of MM against the hyperlipidemic condition and bacterial infections. Moreover, scientific research indicates that similar species from different environmental and geographical locations significantly vary in their metabolites and biological activities [20]. Finally, we were engrained to manifest the concealed medicinal properties of *Melastoma malabathricum* Linn (MM) shoot and flower against the hyperlipidemic condition through a mice model.

## Methods

### Plant materials

The shoot and flower (purple-magenta petals) of *Melastoma malabathricum* were collected from the local area near Tetulia Sub-district of Panchagarh in Bangladesh and kept in a shading place. Subsequently, the plant is authenticated at the Department of Biotechnology and Genetic Engineering, Mawlana Bhashani Science and Technology University (MBSTU), Santosh, Tangail-1992, Bangladesh.

### Preparation of extracts

The shoots were first appropriately washed with clean water to remove any adhering dirt. The lush flowers were also collected, and both of the plant materials had been kept in a shady ambiance. Then, completely dried, both plant materials were ground into a coarse powder by a grinding machine and stored in an airtight container. The crude powder of *Melastoma malabathricum* was dissolved in three solvents (1:5 g/ml), ethanol, acetone, and water, respectively, in different cylinders and enshrouded with aluminium foil for 7 days with occasional shaking and stirring to make the plant extract. Whether the shoot powder had given respectfully into absolute ethanol, water, and the flower powder had also dissolved only into acetone. Subsequently, each solution of plant powder was filtered through Whatman No.1 filter paper, and the filtrate solvents were completely evaporated under reduced pressure at 40 °C using a rotary evaporator. Subsequently, the derived plant extracts were tagged as MM shoot ethanol (MSE) extract, MM shoot water (MSW) extract, and MM flower acetone (MFA) extract. Finally, the plant extracts were kept in small sterile bottles under refrigerated conditions (4 °C) until used.

### Preparation of high-cholesterol diet (HCD)

The 80 g cholesterol powder was mixed finely with 500 ml vanaspati ghee and edible coconut oil, resulting in a 160 mg/ml concentration, where the vanaspati ghee and palatable coconut oil ratio was 3:2 (v/v) [21]. The high-fat diet was supplied with daily grower feeds (Sonali grower feed in Bangladesh) during the induction of hyperlipidemic mice.

### Preparation of atorvastatin solution

5 mg tablets (*n* = 20) were obtained from a renowned dispensary at Tangail, Bangladesh, and finely pulverized before being dissolved in 1 ml distilled water to give a concentration of 5 μg/μl. An oral dose was chosen and provided a fixed time each day based on body mass.

### Preparation of carbimazole solution

5 mg tablets (*n* = 20) were collected from the same dispensary, ground and finely mixed in distilled water to a rising concentration of 5 μg/μl. They were administered orally every day during the HCD mediated induction of hyperlipidemic mice.

### Animals handling

Abiding the ethical rules, the female albino 30 mice (~ 25 g) were imported from the international Center for diarrhoeal disease research, Bangladesh (ICDDR, B) and taken care of in well-ventilated mice laboratory at the Department of Biotechnology and Genetic Engineering, MBSTU. The age of the mice was 2 months, and they were given 5 days to acclimatize with this milieu, where they were exposed to 12 hours of light and 12 hours of darkness. They were provided grower feeds with clean tap water once daily (8.00 am - 9.00 am).

### Induction of hyperlipidemia

The experiment was conducted on two-month-old female mice, where all of the groups were indiscriminately given the daily grower feeds and potable water. But, except for group A, the rest of the groups went through a high cholesterol diet (HCD) with 1.35 mg/kg of carbimazole for 3 weeks to induce hyperlipidemic mice. The hyperlipidemic condition of the HCD mice had confirmed taking weight compared with the standard control that was prominent after 3 weeks.

### Experimental design

Thirty albino female mice had been categorized into six groups in which each of the groups had five mice (~ 25 g) to commence the experiment with potable water to drink.

Group A: (Normal Control): Solely daily grower feed was provided.

Group B: (Hyperlipidemic control): High cholesterol diet (HCD) 2500 mg/kg and 1.35 mg/kg of carbimazole were given with neither drug nor extract.

Group C: Provided HCD 2500 mg/kg, 1.35 mg/kg of carbimazole, 500 mg/kg MFA dose.

Group D: Served HCD 2500 mg/kg, 1.35 mg/kg of carbimazole, 250 mg/kg MSE dose.

Group E: Served HCD 2500 mg/kg, 1.35 mg/kg of carbimazole, 500 mg/kg MSE dose.

Group F: (Positive control): Received HCD 2500 mg/kg, 1.35 mg/kg of carbimazole and 20 mg/kg of atorvastatin.

The treatment of this experiment was persistent for 28 days, where three groups received different plant extracts with different concentrations, and the impacts of these doses had evaluated by quantifying the liver markers (AST, ALT, and ALP) and performing histopathology.

### Biochemical assay and histopathology

Given 28 days of treatments, all groups were anesthetized with chloroform and immediately sacrificed to collect the blood from the heart using the syringe. Subsequently, blood was kept in the Eppendorf tube and placed on an icebox to be clotted, and some organs (Heart, Liver, Kidney) were preserved in 10% formaldehyde for histopathology analysis [22]. The clotted blood was centrifuged at 4000 rpm at 4 °C for 5 min to obtain serum which is then used to identify cardiovascular and liver-damaging markers using the “Human diagnostics worldwide kit” according to manufacturer’s instructions where Bioanalyzer (Humolyzer 3000, Germany) was utilized. Among the markers, the magnitude of LDL and VLDL had been calculated based on Friedewald et al. formula [23]: (a) VLDL = TG/5 (mg/dl) (b) {LDL = TC – (HDL + TG)} (mg/dl).

The histopathology was performed amid glass slide preparation, where organs were dehydrated by passing through a graded series of alcohol and embedded in paraffin blocks to prepare 5 mm sections using a semi-automated rotary microtome. These slides were stained using hematoxylin and eosin [24].

### Antibacterial potentiality

#### Gram-negative bacteria

The pathogenicity of gram-negative bacteria is immensely harmful to human health and other animals. The prevalence of diarrhea due to *Shigella* and *E.coli* in Bangladesh is prominent [25, 26]; that’s why some strains of these species had taken to bring out the antibacterial potentiality of *Melastoma malabathricum* using MSE and MSW Extract. The name of these gram-negative bacterial strains is *Enterotoxigenic E.coli* (ETEC), *Enteropathogenic E.coli* (EPEC), *Shigella boydii* (SB), *Shigella flexneri* (SF), *Shigella sonnei* (SS), *Shigella dysenteriae* (SD).

##### Disk diffusion method

The disk diffusion method was used to ferret out the antibacterial potentiality of MSE and MSW extract using Mueller Hinton Agar (MHA). First, the young culture of all the above strains was prepared by transferring 15 μl suspension culture to Mueller Hinton Broth (MHB) for 2.30 h of incubation at 37 °C. Afterward, 100 μl of young culture were soaked in an MHA plate and impregnated with sterile filter paper disks (6 mm in diameter). Subsequently, different concentrations of MSE and MSW extracts were dissolved in 5% dimethyl sulfoxide (DMSO), and 15 μl of each extract was placed on the paper disks (600 μg/disc, 900 μg/disc, 1200 μg/disc, 1500 μg/disc) and standard antibiotics (Chloramphenicol 30 μg/disc) as well. Finally, all cultured plates were incubated at 37 °C overnight to give proper ambiance to grow bacterial culture. During that moment, contingent on phytochemicals concentration, the growth of bacterial culture was obviated around these disks that exhibited a transparent zone called the zone of inhibition. The zone of inhibition is measured with mm units.

### Statistical analysis

All of the results in our current experiment were manifested as mean ± standard error mean (SEM), the analysis of variance (ANOVA) was utilized to demonstrate significant differences among the independent groups (IBM SPSS Statistics, Version 25). The values were significantly considered when the *p*-value was < 0.05.

## Result

### Acute toxicity study

The pre-existing research on MM ethanolic extract has unveiled that these medicinal plants haven’t had any acute toxicity impact in the animal model. The experimental dose was 2000 mg/kg, and 5000 mg/kg continued for experimenting. No noticeable change occurred in skin and behavior like diarrhea, sleeping, salivation, and tremors [27].

We conducted our experiment with two plant extracts at a dose of 250 mg/kg and 500 mg/kg for 28 days; no mice demise, phenotypic and behavioral changes occurred due to extract toxicity. This result was also substantiated further by histopathology analysis of the liver, kidney, heart.

### The consequence of MM plant extracts on liver

In our current experiment, the expression of liver markers was elevated significantly (*P* < 0.001) in the HCD induced hyperlipidemic mice compared with the normal control group. At the end of the treatment, the liver enzyme expression was tremendously reduced (P < 0.001, *P* < 0.01) in groups C, D, and E using fixed doses, compared with group B (Table 1). The magnitude of reduction of the liver enzyme expression (ALP, ALT, AST) comparing with hyperlipidemic control group B, were for the doses (a) MFA-500 mg/kg: about 7%, 24%, 17% (b) MSE-250 mg/kg: about 5%, 15%, 8% and (c) MSE-500 mg/kg: about 30%, 33%, 24% and, in the group C, D and E, respectively. On the other hand, group F, treated with atorvastatin (Drug) 20 mg/kg, demonstrated a significantly higher (*P* < 0.001) level of AST, ALT, and ALP expression than group B (Table 1).

**Table 1:** Relation between different conc. of MM plant extracts and drug to livers

Liver markers (U/L)	Group A (Normal control)	Group B (HYPER control)	Group C (MFA-500 mg/kg)	Group D (MSE-250 mg/kg)	Group E (MSE-500 mg/kg)	Group F (ATOVAT-20 mg/kg)
ALP	132.2 ± 1.50	198.8 ± 1.16^a^	185.4 ± 1.81^d^	189.4 ± 1.72^e^	139.4 ± 1.86^d^	260.4 ± 1.44^d^
ALT	48 ± 1.58	102.4 ± 1.21^a^	79.2 ± 1.32^d^	87.2 ± 1.50^e^	69.2 ± 1.59^d^	168.6 ± 1.57^d^
AST	189 ± 1.58	275.2 ± 2.03^a^	228.6 ± 1.63^d^	258 ± 1.70^d^	210.4 ± 1.94^d^	388.8 ± 2^d^

Outcomes of this experiment is expressed as mean ± standard error of mean (SEM) where n = 5; ^a^P < 0.001; compared with normal control; ^d^P < 0.001; ^e^P < 0.01; compared with hyperlipidemic control; One-Way ANOVA: Dunnett’s multiple comparison test.

**Fig. 1:**
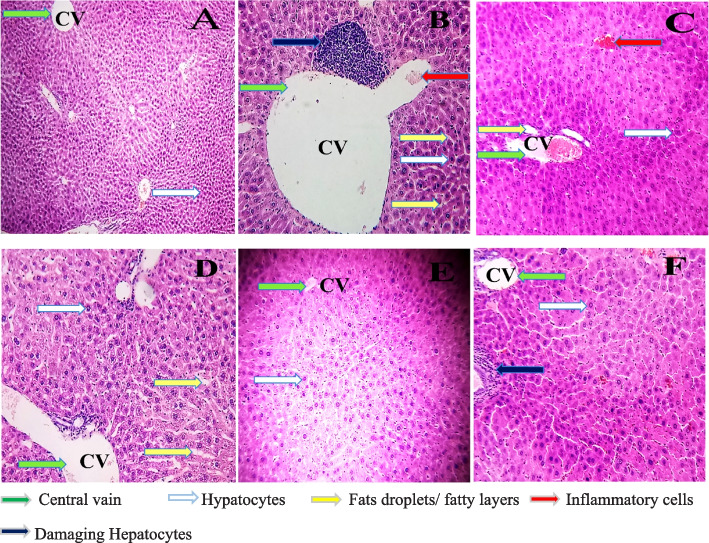
The Potentiality of *Melastoma malabathricum* extracts to protect the liver in HCD mice. **a** Normal group: Demonstrated no fat accumulation and hepatocytes enlargement (gray arrow). **b** Hyperlipidemic control: Exhibited prominently amassed micro fatty layers (yellow allow), enlarged hepatocytes (gray arrow), and cellular damaging (blue arrow) surrounding the central vein (CV) (green arrow) but also noticed the presence of inflammatory cells in the CV (red arrow). **c** MFA-500 mg/kg: Deposition of fat droplets among the intercellular space (yellow arrow), inflammatory cells infiltration (red allow), and,  a little bit larger hepatocytes were also observed (gray arrow). **d** MSE-250 mg/kg: Fat granules hoarded among the intercellular space (yellow arrow) and hepatocyte enlargement (gray arrow) also occurred around CV. **e** MSE-500 mg/kg: Cellular infrastructure was almost the same as a normal control group, with no fat globules or fatty layer found in the liver. **f** ATOVAT-20 mg/kg: Seemingly no sign of deposited fats but a bit of cellular deformation (blue arrow) observed like as group D. (Pictures magnification 20X)

Histopathology was done to substantiate the biochemical test of liver-damaging markers. The metabolic conversion of xenobiotics, phytochemicals, and deposited fats in the liver is converted into reactive intermediates such as electrophilic compounds or Reactive oxygen species (ROS), which can potentially transmute the structure and function of cellular macromolecules [28]. The group F treated with ATOVAT-20 mg/kg demonstrated scarring, which might be due to oxidative stress originating (ROS) from atorvastatin metabolism and amassed fats in the liver. In contrast, group C treated with MFA-500 mg/kg also didn’t restrain the deposition of fats significantly. Even though group D was administered MSE-250 mg/kg had a little bit of liver scarring and deposited fats, in group E, using MSE-500 mg/kg dose declined deposition of fats in the liver that was almost similar to normal control liver (Fig. 1).

### The consequence of MM plant extracts on lipid profiling

In the hyperlipidemia-induced mice, TG, TC, VLDL, and LDL level was significantly higher (*P* < 0.001), whereas HDL level was prominently lower (P < 0.001) compared with the normal group. During treatment, group C administered with MFA-500 mg/kg had shown trivial reduction (*P* < 0.01) of TG, TC, VLDL and LDL and elevated paltry (*p* > 0.05) HDL level; whereas group D, E and F with MSE-250 mg/kg, MSE-500 mg/kg and ATOVAT-20 mg/kg declined significantly (*P* < 0.001) and HDL level also augmented (P < 0.001; *P* < 0.05) compared with group B (Table 2).

**Table 2:** The antihyperlipidemic potentiality of MM plant extracts at different conc. in HCD mice

Lipid-molecules(mg/dl)	Group A (Normal control)	Group B (HYPER control)	Group C (MFA-500 mg/kg)	Group D (MSE-250 mg/kg)	Group E (MSE-500 mg/kg)	Group F (ATOVAT-20 mg/kg)
TG	115 ± 1.58	225.8 ± 1.80^a^	218.2 ± 1.37^e^	160.6 ± 1.72^d^	131.8 ± 1.56^d^	123 ± 1.41^d^
TC	50 ± 1.58	110 ± 1.64^a^	107.6 ± 1.63^e^	78.2 ± 1.65^d^	62.8 ± 1.36^d^	55.8 ± .97^d^
HDL	23 ± 1.58	10.4 ± 1.72^a^	13.4 ± 1.29^hn^	16.6 ± 1.43^f^	22.4 ± 1.81^d^	17.4 ± 1.36^f^
VLDL	23 ± .32	45.1 ± .36^a^	43.6 ± .27^e^	32 ± .32^d^	26.4 ± .33^d^	24.6 ± .28^d^
LDL	4 ± .47	54.4 ± 3^a^	39.8 ± 8.95^e^	29.5 ± .71^d^	14 ± 3.45^d^	13.8 ± .76^d^

Outcomes of this experiment is expressed as mean ± standard error of mean (SEM) where *n* = 5; ^a^P < 0.001; compared with normal control and ^d^P < 0.001; ^e^P < 0.01; ^f^P < 0.05; ^hn^p > 0.05 compared with hyperlipidemic control.One-Way ANOVA: Dunnett’s multiple comparison test.

### The consequence of MM extracts on the heart

**Fig. 2:**
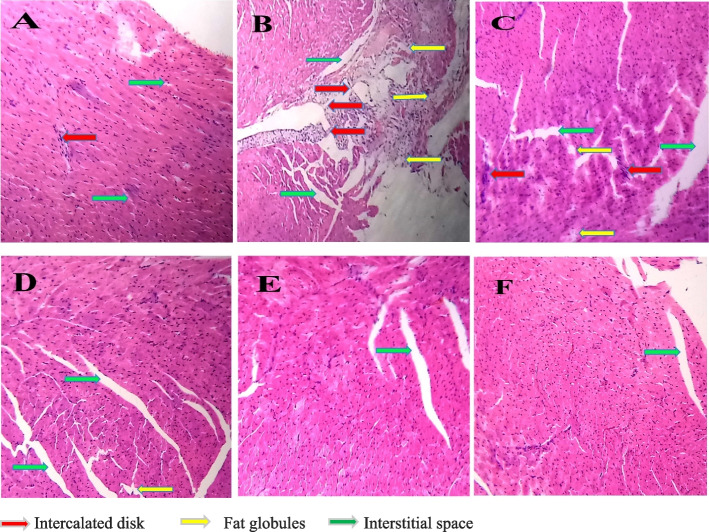
Effect of *Melastoma malabathricum* plant extracts at different conc. on the heart in HCD mice. **a** Normal group: Histopathology of the normal group’s heart demonstrated no changes among the intercalated disk (red arrow) and interstitial space (green arrow). **b** Hyperlipidemic control: Intercalated disk was contused (red arrow), interstitial space was augmented (green arrow), and cellular deformation was induced due to infiltration of fat molecules (yellow arrow). **c** MFA-500 mg/kg: Intercalated disk was deteriorated (red arrow), as well as the interstitial space was ameliorated (green arrow) endorsed by amassing fat globules (yellow arrow). **d** MSE-250 mg/kg: Histopathology of this group’s exhibited enhanced interstitial space without any cellular contortion. A little bit presence of fats was discerned in this analysis (yellow arrow). **e** MSE-500 mg/kg: This dose showed a stunning histopathology result which was almost similar to normal heart structure, minimized interstitial space without any cellular contusion. **f** ATOVAT-20 mg/kg: Atorvastatin drug control group’s histopathology appeared no cellular deformation that was almost normal but manifested with trivial interstitial space. (Pictures magnification 20X)

The result of lipid profiling is buttressed by histopathology analysis of the heart of each group of mice. Group B and C exposed cardiac muscle deformation, distorted intercalated disk, and significant hiatus between interstitial spaces due to deposition of fats (Yellow arrow), which have the strong potential to oxidize and trigger the immune response. Alongside, MSE-250 mg/kg dose-treated group D also manifested ample interstitial space and little fat deposition without any cardiac muscle contortion. In contrast, group E treated with MSE-500 mg/kg declined the deposition of fats and cardiac muscle distortion in the heart that was almost congruous with group A and F (Fig. 2).

### The consequence of MM extracts on the kidney

**Fig. 3:**
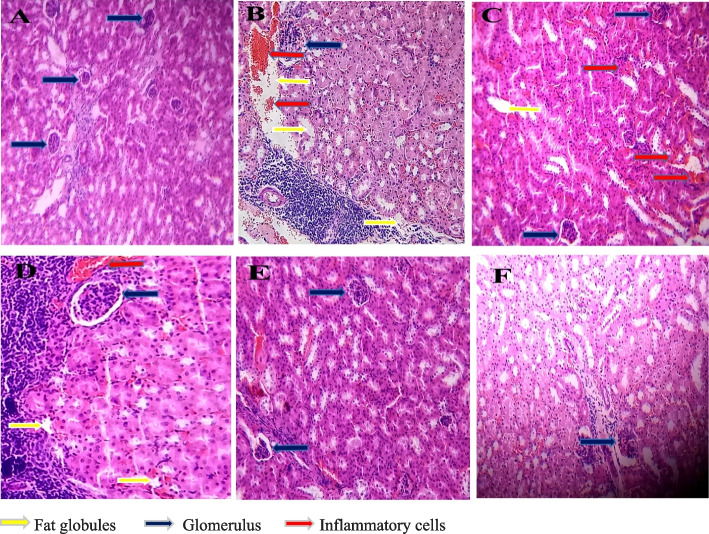
Effect of *Melastoma malabathricum* extracts at different conc. on the kidney in HCD mice. **a** Normal group: Histopathology demonstrated the average size of the glomerulus (blue arrow) without any accumulation of fatty layers or globules. **b** Hyperlipidemic control: Exacerbated and enlarged glomerulus (blue arrow) due to the deposition of fat molecules as well as the presence of inflammatory cells in the blood vessels (red arrow). **c** MFA-500 mg/kg: The glomerulus was a little bit larger (blue arrow), demonstrating the presence of inflammatory cells (red arrow) and enlarged fat globules (yellow arrow) after performing histopathology analysis. **d** MSE-250 mg/kg: A bit expanded glomerular size (blue arrow), some fat globules deposition (yellow arrow), and also appeared minute inflammatory cells in the blood vessel (red arrow). **e** MSE-500 mg/kg: The histopathology of the tested drug with this conc. Exhibited typical infrastructure of glomerulus and no prominent sign of fats deposition. **f** ATOVAT-20 mg/kg: Similar histopathology was observed compared to the normal group. (Pictures magnification 20X)

The kidney histopathology demonstrated the presence of inflammatory cells, amassed fats molecules, and enlarged glomerulus in group B. This horrendous condition manifested maybe because of fat infiltration and subsequently oxidized LDL cholesterol. Group C, treated with MFA-500 mg/kg, exhibited the same expression. On the other hand, group D treated with MSE-250 mg/kg revealed attenuated expression of inflammatory cells, little deposition of fat molecules, and small size of glomerulus compared with hyperlipidemic control. But when the treatment was performed with the tested drug MSE-500 mg/kg, group E demonstrated appreciable results found no inflammatory cells, deposited fats globules, and standard glomerulus size almost resembled group A and F (Fig. 3).

### The trend of weight during treatment

Three weeks later, the body weight in all groups (Provided HCD) had waxed significantly (*P* < 0.001) compared with the normal group, immediately given treatment with MSE-250 mg/kg and MSE-500 mg/kg in groups D and E, resulting abated the rapid upward bodyweight significantly (*P* < 0.01, P < 0.001) at the 6th and 7th weeks (Table 3). But group C treated with the MFA-500 mg/kg manifested a slight tendency (*P* < 0.05) to lose body mass compared with group B.

**Table 3:** The propensity of body weight during treatment with different MM extracts in hyperlipidemic mice

Weeks (g)		Group A (Normal control)	Group B (HYPER control)	Group C (MFA-500 mg/kg)	Group D (MSE-250 mg/kg)	Group E (MSE-500 mg/kg)	Group F (ATOVAT-20 mg/kg)
Induction	1^day^	24.2 ± .07	24.24 ± .09	23.54 ± .07	23.48 ± .09	23.68 ± .07	23.82 ± .05
	1st	26.6 ± .17	27.7 ± .09^a^	26.4 ± .09^a^	26.5 ± .09^a^	26.8 ± .04^a^	26.7 ± .07^a^
	3rd	28.8 ± .05	33.5 ± .09^a^	32.9 ± .04^a^	31.6 ± .09^a^	32.3 ± .12^a^	31.2 ± .09^a^
Treatment	6th	33.6 ± .68	42.7 ± .66^a^	39.4 ± .68^f^	38.7 ± .75^e^	37.4 ± .80^d^	36.7 ± .71^d^
	7th	34.9 ± .70	44.3 ± .72^a^	41.4 ± .65^f^	39.9 ± .69^d^	38.8 ± .69^d^	38.3 ± .66^d^

Results are expressed as mean ± standard error of mean (SEM); where n = 5; ^a^P < 0.001; compared with normal control; ^d^P < 0.001; ^e^P < 0.01; ^f^P < 0.05 compared with hyperlipidemic control. One-Way ANOVA: Dunnett’s multiple comparison test.

### The antibacterial potentiality of MM plant extract

Different concentrations of MSE extract (A = 600 μg/disc, B = 900 μg/disc, C = 1200 μg/disc, D = 1500 μg/disc) had exhibited prominent zone of inhibition against *S. dysenteriae, S. sonnei, S.flexneri* strain, whereas MSW extract demonstrated a mediocre zone of inhibition. But both MSE and MSW extract concentrations revealed less potentiality to inhibit the growth of *S. boydii*, *ETEC,* and *EPEC* strains (Table 4).

**Table 4:** The zone of inhibition of different conc. of 2 MM extracts on some pathogenic bacteria

Zone of Inhibition(mm in diameter)										
Name of the Bacteria	MSE				MSW				NC	CP
	[A]	[B]	[C]	[D]	[A]	[B]	[C]	[D]	[E]	[F]
*S. dysenteriae*	13 **±** 1	14.75 **±** .25	15.50 **±** .50	16.50 **±** .50	8.50 **±** .50	9 **±** 1	9 **±** 1	9.50 **±** .50	0	26 **±** 1
*S. sonnei*	9.50 **±** .50	10.50 **±** .50	10.50 **±** .50	11 **±** 1	7 **±** 1	8.50 **±** .50	8.50 **±** .50	8.50 **±** .50	0	24.50 **±** .50
*S.flexneri*	11 **±** 1	11.50 **±** .50	12.50 **±** .50	13.50 **±** .50	6.50 **±** .50	7.50 **±** .50	7.50 **±** .50	8.50 **±** .50	0	21.50 **±** .50
*S. boydii*	7.50 **±** .50	8.50 **±** .50	9 **± 1**	10.50 **±** .50	6.50 **±** .50	7.50 **±** .50	7.50 **±** .50	8.50 **±** .50	0	24.50 **±** .50
*ETEC*	6.50 **±** .50	8 **± 0**	8.50 **±** .50	9 **± 0**	6.50 **±** .50	7 **± 0**	7.50 **±** .50	8 **± 0**	0	24.50 **±** .50
*EPEC*	6.50 **±** .50	8 **± 0**	8.50 **±** .50	9 **± 0**	6 **± 0**	7 **± 0**	7 **± 0**	8 **± 0**	0**±**	16.50 **±** .50

Results are expressed as mean ± standard error of mean (SEM) where n = 2; A = 600 μg/disc; B = 900 μg/disc; C = 1200 μg/disc; D = 1500 μg/disc; E = 15 μL/disc of 5% DMSO; F = 30 μg/disc of Chloramphenicol (CP); Negative control (NC); MSE = *Melastoma malabathricum* shoot ethanol extract; MSW = *Melastoma malabathricum* shoot water extract.

## Discussion

The *Melastoma malabathricum* has tremendous medicinal significance in several disease conditions like diarrhea, arthritis, gastric ulcer, skin disorder, cancer, diabetics, and high blood pressure because of possessing profuse phytochemicals [19].

The major concern of our study was to investigate the comparative anti-hyperlipidemic potency of MSE and MFA extract using two solvents. This approach has proceeded in hyperlipidemia-induced mice with a high cholesterol diet and carbimazole [29]. The HCD was prepared by combining miscellaneous fat-rich products like cholesterol, coconut oil, and natural Ghee [30]. On the other hand, Carbimazole (pro-drug) is an antagonist of the thyroid peroxidase enzyme; therefore, T3 and T4 thyroid hormones are reduced. Furthermore, it has been recorded that during hypothyroidism, the total blood cholesterol, VLDL, and TG level is ameliorated; that’s why this tactic had been taken to rapidly increase the blood total cholesterol level in the mice [31, 32].

The hyperlipidemic condition was induced by ingesting HCD and carbimazole to all groups with their daily feeds except the normal control according to their body mass. At every single week, the weight was recorded (Table 3), whereas the weight was voluminous compared with the normal control in the last week. It is also well defined that increasing cholesterol levels such as TG, TC, HDL, VLDL, and LDL propel body mass [33]. Since the discrepancy of body weight of these groups was ameliorated compared with normal control, the level of the lipid molecules was elevated in the blood that had been substantiated performing lipid profiling (Figs. 5 and 6). After absorption, the flush of lipid molecules in the blood was amassed in the liver, heart, and kidney that had manifested by histopathology analysis (Figs. 1, 2, and 3). The stockpiling of fat in these organs is baneful due to engender several maladies. For instance: The accumulation of fat globules in the liver instinctively can create scarring, fibrosis, hepatic insulin resistance, and oxidative stress resulting in inflammation due to activation of immune cells such as macrophages, neutrophils as well as inflammatory cytokines (IL-1α/β, TNF- α) [34].

**Fig. 4:**
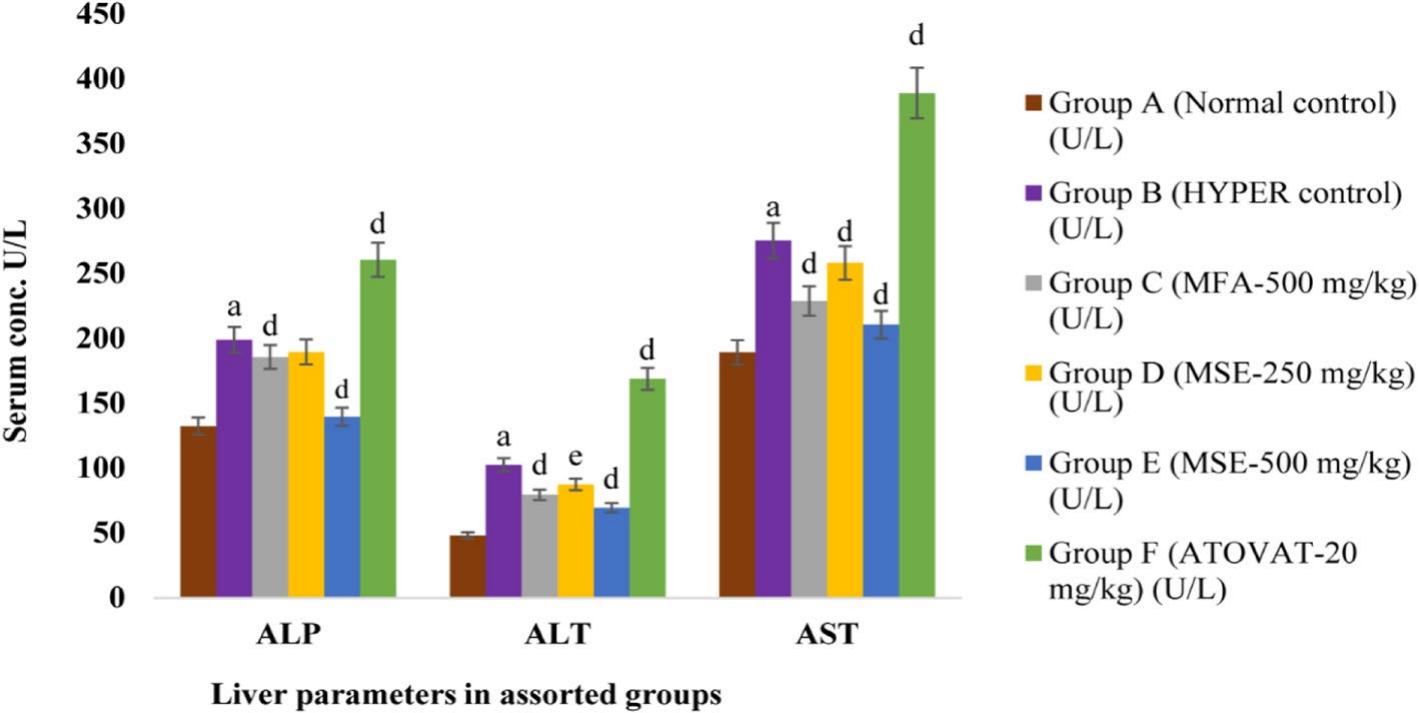
Comparative expression of liver markers at different conc. of MM extracts in HCD mice. The expression of these markers are mean ± standard error of mean (SEM); where *n* = 5; ^a^P < 0.001; compared with normal control; ^d^P < 0.001; ^e^P < 0.01; compared with hyperlipidemic control

In our experiment in group B, the liver condition was exacerbated due to the deposition of fat droplets that could produce oxidative stress and inflammatory cytokines, which demolished the surrounding cells resulting in the liver enzyme’s expression being augmented in the blood serum (Fig. 4). The drug control group demonstrated excessive AST, ALT, ALP levels than the other groups (Table 1); this extensive magnitude could be because of injured liver cells through ROS during xenobiotics (Atorvastatin) metabolism [28]. On the other hand, groups C, D, and E with the treatment of MM plant extract (MFA-500 mg/kg, MSE-250 mg/kg, MSE-500 mg/kg) exhibited less expression of these markers, when in group B, the manifestation of these enzymes was prodigious (Fig 4). In our current experiment, we observed that the MSE-500 extract had a magnificent effect to protect liver deposition of fats and scarring after 28 days of treatment in group E. In contrast, groups B, C, D, and F were present either fat deposition or scarring (Fig. 1).

**Fig. 5:**
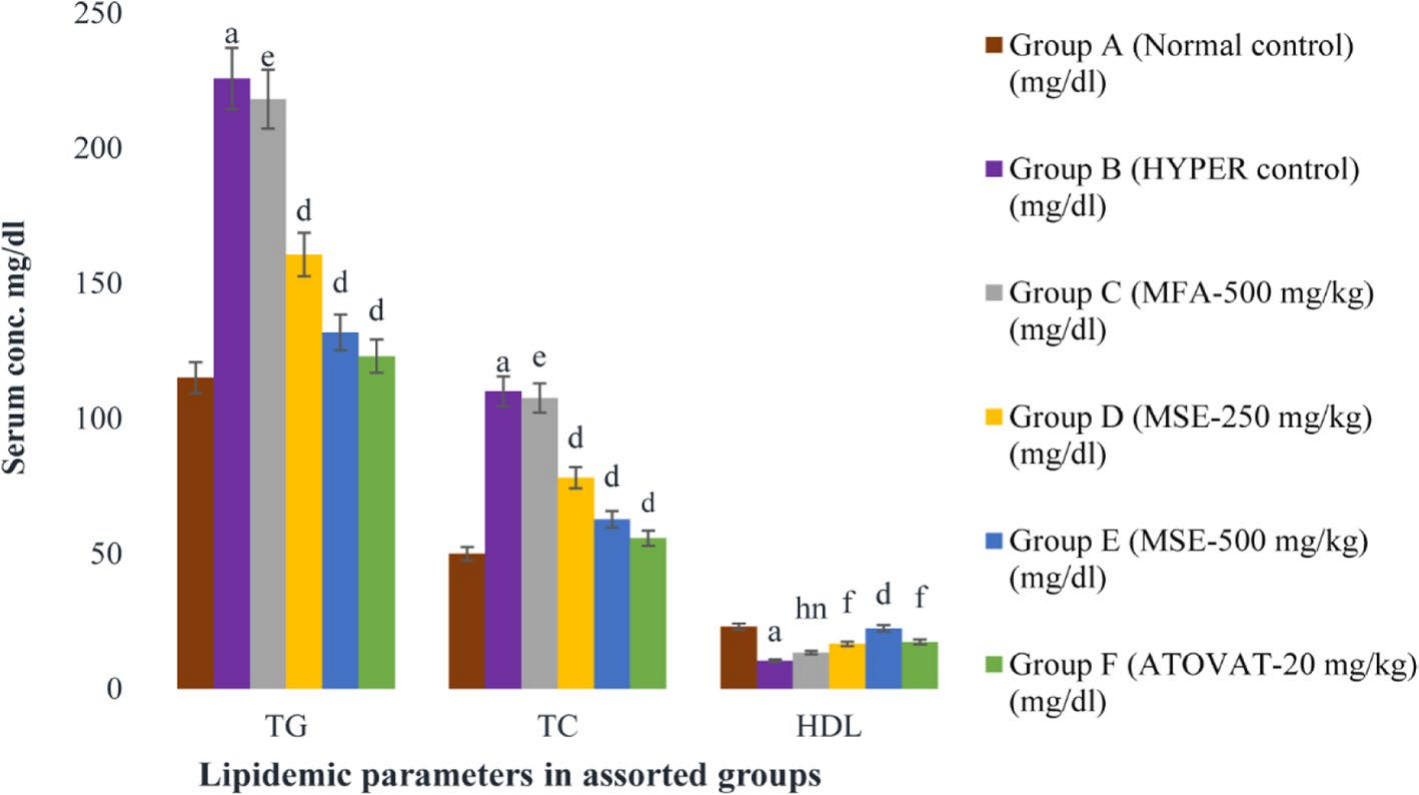
Comparative expression of TG, TC and HDL at different conc. of MM extracts in HCD mice. The expression of these markers are mean ± standard error of mean (SEM); where *n* = 5; ^a^P < 0.001; compared with normal control; ^d^P < 0.001; ^e^P < 0.01; ^f^P < 0.05; ^hn^p > 0.05 compared with hyperlipidemic controls

**Fig. 6:**
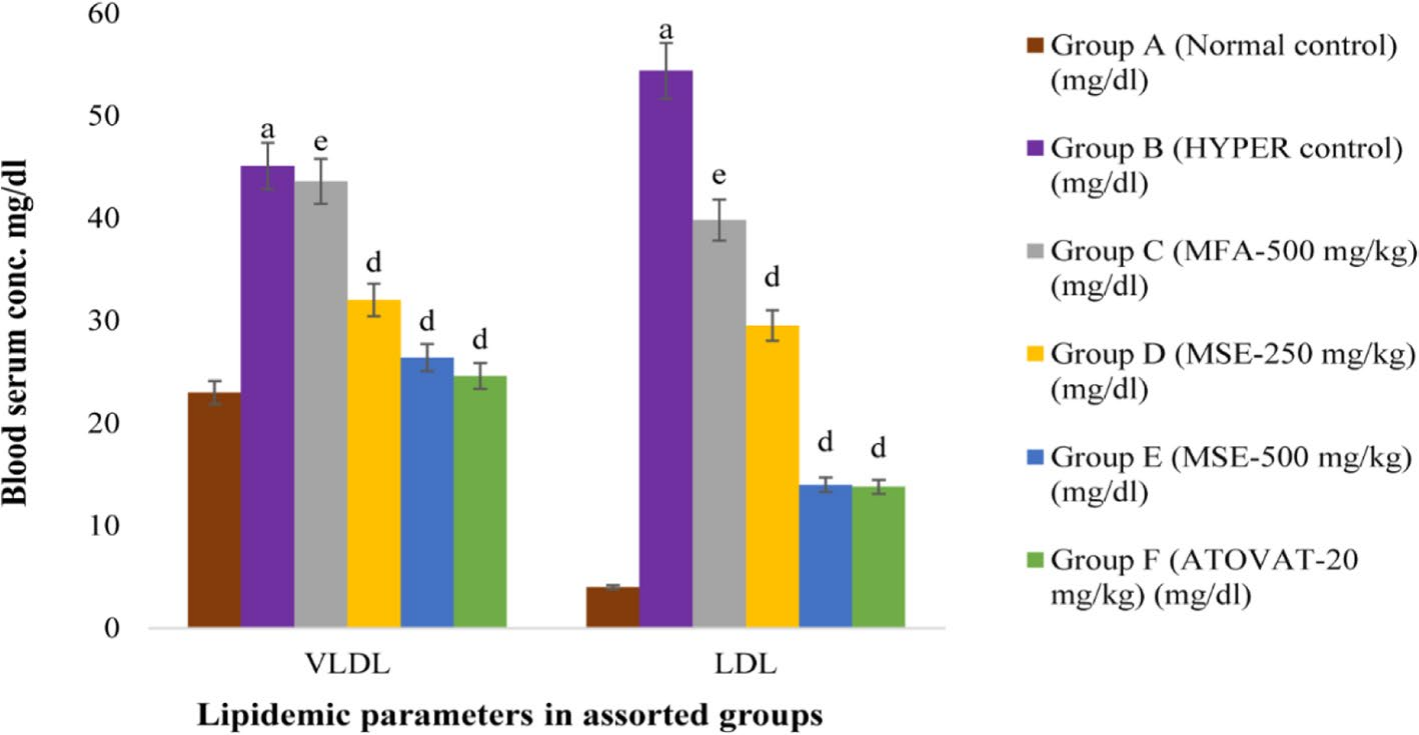
Comparative expression of VLDL and LDL of MM extracts at different conc. in HCD mice. The expression of these markers are mean ± standard error of mean (SEM); where n = 5; ^a^P < 0.001; compared with normal control; ^d^P < 0.001; ^e^P < 0.01; compared with hyperlipidemic control

In the circulatory system, the plethora of LDL cholesterol reaches sub-endothelial space through the surface adhesion molecules due to endothelial dysfunction and becomes so tendentious to oxidize LDL. Subsequently, the macrophage is activated to engulf the oxidized LDL and release pro-inflammatory cytokines, which engenders an inflammatory environment that contributes to plaque formation in the blood vessel for the impairment of blood flow [35, 36]. That’s why the presence of much LDL in the bloodstream is baneful for the body’s vascular function. Our current study has manifested the anti-hyperlipidemic efficiency of orally administrated different conc. of MM plant extract such as MFA-500 mg/kg exhibited trivial potency (4%, 3%, 5% and 28%) to subtract the lipid molecules (TG, TC, VLDL, LDL) respectively, but MSE-250 mg/kg lessened more (28%, 30%, 29% and 47%), whereas the MSE-500 mg/kg tested as alternative drug, had declined tremendously (41%, 44%, 43% and 74%) comparing with drug control (Figs. 5 and 6). Concomitantly, HDL level augmented (30%, 60% and 70%) using MFA-500 mg/kg, MSE-250 mg/kg and ATOVAT-20 mg/kg but administered MSE-500 mg/kg endorsed more to flush HLD up to 120% (Table 2 and Fig. 5). Afterward, the histopathology analysis elicited that the MSE-500 mg/kg extract could effectively restrain fats deposition and keep normal cellular infrastructure in the heart (Fig. 2).

The kidney commonly excretes xenobiotics after metabolic biotransformation even though atorvastatin excretion is almost all biliary, less than 2% eliminated through the kidney [37]. Phytochemicals are xenobiotics; that’s why both of them should have a significant impact on kidneys that had been manifested by kidney histopathology for all groups after 28 days of treatment. Our recent study revealed that in groups C and D using MFA-500 mg/kg and MSE-250 mg/kg, the kidney condition was least aggravated, such as inflammatory blood cells and deposition of fats compared with group B (Fig. 3). Whereas in groups E and F using MSE-500 mg/kg and ATOVAT-20 mg/kg, the presence of inflammatory blood vessels and fat deposition around the glomerulus disappeared, almost congruous with normal kidneys’ phenotype.

Alongside the effect of MM extracts in blood cholesterols, livers, kidneys, and hearts in hyperlipidemic mice, the antibacterial activity was also be checked against some gram-negative pathogenic bacteria using four concentrations of MSE and MSW. The maximum zone of inhibition of MSE extract was 17 mm against *S. dysenteriae*, 14 mm against *S. flexneri*, and 12 mm against *S. sonnei.* Moreover, the maximum zone of inhibition of MSW extract was 10 mm against only *S. dysenteriae* (Table 4)*.* In contrast, *ETEC* and *EPEC* strain demonstrated paltry potency with all concentrations of these two extracts.

## Conclusion

In our current inquisition, the MSE-250 mg/kg dose demonstrated significant results in all cases. However, treatment with MSE-500 mg/kg was appealing and exposed its anti-hyperlipidemic potency by increasing HDL level. Moreover, this dose was liver and kidney-friendly by protecting inflammation and fat deposition, almost comparable to atorvastatin. But the stunning matter is that after 28 days of treatment, group F noticed a glimpse of liver scarring but didn’t exhibit anything like this in group E, which hints at using MSE-500 mg/kg as an alternative drug during the hyperlipidemic condition. On the contrary, the MFA-500 mg/kg didn’t exhibit any anti-hyperlipidemic potency after 28 days of treatment but showed a slightly amicable relationship with the liver. Alongside, the MSE extract at 1500 μg/disc exhibited a maximum zone of inhibition against most of the pathogenic strain than the MSW extract during the current experiment.

### Limitation

The phytochemical screening and antioxidant properties of MM extracts didn’t carry out our fundamental research that would make it stronger and precise to demonstrate why extracts are friendly for liver, kidney, heart and which phytochemicals are responsible for antibacterial.

## Data Availability

The dataset analyzed during our current study is available from the corresponding author on reasonable request.

## Abbreviations

MM: *Melastoma malabathricum* Linn
HCD: High cholesterol diet
MSE: MM shoot ethanol
MSW: MM shoot water
MFA: MM flower acetone
ATOVAT: Atorvastatin
AST: Aspartate aminotransferase
ALT: Alanine aminotransferase
ALP: Alkaline phosphatase
TC: Total cholesterol
TG: Triglyceride
LDL: Low-density lipoprotein
VLDL: Very low-density lipoprotein
CD: cardiovascular disease
T2DM: diabetes mellitus type 2
PAD: peripheral arterial disease
CHD: coronary heart disease
ROS: Reactive oxygen species
AD: Alzheimer disease
ETEC: *Enterotoxigenic E.coli*
EPEC: *Enteropathogenic E.coli*
SB: *Shigella boydii*
SF: *Shigella flexneri*
SS: *Shigella sonnei*
SD: *Shigella dysenteriae*
MHA: Muller Hinton Agar
MHB: Mueller Hinton Broth
DMSO: Dimethyl sulfoxide
HYPER: Hyperlipidemic
